# NS1 Antigenemia and Viraemia Load: Potential Markers of Progression to Dengue Fatal Outcome?

**DOI:** 10.3390/v10060326

**Published:** 2018-06-14

**Authors:** Priscila Conrado Guerra Nunes, Rita Maria Ribeiro Nogueira, Manoela Heringer, Thaís Chouin-Carneiro, Cintia Damasceno dos Santos Rodrigues, Ana Maria Bispo de Filippis, Monique da Rocha Queiroz Lima, Flávia Barreto dos Santos

**Affiliations:** 1 Viral Immunology Laboratory (LIV), Oswaldo Cruz Institute—FIOCRUZ, Avenida Brasil, 4365. Manguinhos, Rio de Janeiro 21040-360, Brazil; pricgn@ioc.fiocruz.br (P.C.G.N.); manoela.heringer@ioc.fiocruz.br (M.H.); tatachouin@gmail.com (T.C.-C.); moniqueq@ioc.fiocruz.br (M.d.R.Q.L.); 2 Flavivirus Laboratory (LABFLA), Oswaldo Cruz Institute—FIOCRUZ, Avenida Brasil, 4365. Manguinhos, Rio de Janeiro 21040-360, Brazil; rita@ioc.fiocruz.br (R.M.R.N.); cintia.damasceno@ioc.fiocruz.br (C.D.d.S.R.); abispo@ioc.fiocruz.br (A.M.B.d.F.); 3 Hematozoa Transmittors Mosquitoes Laboratory, Oswaldo Cruz Institute, Rio de Janeiro 21040-360, Brazil

**Keywords:** dengue, fatal cases, NS1 antigenemia, viraemia load

## Abstract

Dengue is a worldwide problem characterized by a multifactorial pathogenesis. Considering the viral components, it is known that high viremia or high levels of the secreted nonstructural protein 1 (NS1) may be associated with a more severe disease. We aimed to characterize the NS1 antigenemia and viremia in dengue fatal and non-fatal cases, as potential markers of progression to a fatal outcome. NS1 antigenemia and viremia were determined in Brazilian dengue fatal cases (*n* = 40) and non-fatal cases (*n* = 40), representative of the four dengue virus (DENV) serotypes. Overall, the fatal cases presented higher NS1 levels and viremia. Moreover, the fatal cases from secondary infections showed significantly higher NS1 levels than the non-fatal ones. Here, irrespective of the disease outcome, DENV-1 cases presented higher NS1 levels than the other serotypes. However, DENV-2 and DENV-4 fatal cases had higher NS1 antigenemia than the non-fatal cases with the same serotype. The viremia in the fatal cases was higher than in the non-fatal ones, with DENV-3 and DENV-4 presenting higher viral loads. Viral components, such as NS1 and viral RNA, may be factors influencing the disease outcome. However, the host immune status, comorbidities, and access to adequate medical support cannot be ruled out as interfering in the disease outcome.

## 1. Introduction

The incidence of dengue has increased dramatically around the world in recent decades, and an estimated 50–100 million cases occur annually [1]. The disease has a broad clinical spectrum ranging from a self-limiting disease in most individuals to a potentially fatal one [2,3]. Fatal cases may occur in over 10% of the severe cases, with 90% of deaths occurring in children under 15 years of age [4]. However, in recent decades, dengue and severe dengue have become more frequent among adults [5].

In Brazil, dengue has become a major public health problem since the 1980s, and it has accounted for 60–80% of the cases reported in the Americas [6,7,8,9]. The co-circulation of four serotypes and the extensive epidemics occurring in Brazilian territory have led to an increased risk of severe and fatal cases [10,11,12,13].

Dengue virus (DENV), a single-stranded positive-sense RNA virus, belongs to the *Flavivirus* genus and *Flaviviridae* family and is classified into four antigenically distinct serotypes (DENV-1 to 4). The DENV nonstructural protein 1 (NS1) is a highly conserved specific soluble glycoprotein that plays a role in viral replication [14] and can be detected in patients’ serum up to 18 days after the onset of symptoms such as a fever [15], with a peak sensitivity in the first three days of fever [16,17].

Several risk factors for a severe disease have been determined, including exposure to a heterologous DENV serotype, infection by certain viral strains and serotypes, age, gender, and some host genetic variants. High DENV load or secreted NS1 levels have been associated with a more severe disease in endemic populations [18,19,20,21,22], as NS1 is involved in vascular leakage and endothelial hyperpermeability by disrupting the endothelial glycocalyx independently of inflammatory cytokines [23,24,25,26]. Here, we sought to characterize the NS1 antigenemia and viremia in dengue fatal cases in comparison to non-fatal ones, representative of the four serotypes, that presented during epidemics that occurred in Brazil.

## 2. Materials and Methods

### 2.1. Ethics Statement

The dengue suspected fatal cases analyzed in this study belong to a collection previously gathered from a Project in the Laboratory of Flavivirus, Oswaldo Cruz Institute, FIOCRUZ, approved (13 May 2014) by resolution number CSN196/96 from the Oswaldo Cruz Foundation Ethical Committee in Research (CEP 274/05), Ministry of Health, Brazil.

### 2.2. Dengue and Fatal Dengue Cases

Non-fatal dengue and dengue fatal suspected cases were received between January 1990 and December 2013 during an active surveillance program performed by the Laboratory of Flavivirus, IOC/FIOCRUZ, Regional Reference Laboratory for the Brazilian Ministry of Health, sited in Rio de Janeiro. In this study, samples were selected according to their availability, volume, case confirmation as dengue based on the WHO criteria [3], serotype, and signs and symptoms. Non-fatal dengue cases presented mild dengue symptoms and were classified as dengue and dengue without alarm signs [3]. A dengue case was classified as fatal when a dengue suspected fatal case was confirmed as such through a laboratory analysis. No DENV co-infections were identified, and the patients’ ages varied from 8 to 80 years old.

DENV infections on non-fatal and fatal cases were analyzed and confirmed by using a laboratorial diagnosis, as follows. Serum samples (up to 8 days after the onset of symptoms) were stored at −70 °C and submitted to virus isolation, molecular methods (reverse transcriptase polymerase chain reaction (RT-PCR), Real-time Reverse Transcriptase PCR (TaqMan) assay (qRT-PCR), NS1 antigen capture ELISA, immunoglobulin M (IgM) antibody-capture MAC-ELISA, and immunoglobulin G (IgG) antibody-capture ELISA (IgG-ELISA) tests. Briefly, for virus isolation and DENV serotyping, the samples were inoculated into C6/36 Aedes albopictus cell line [27] and the serotypes were identified by indirect fluorescent antibody test [28]. An in-house MAC-ELISA was carried out for dengue cases confirmation as described by Nogueira [29]. Alternatively, the Panbio dengue IgM Capture ELISA (Panbio Diagnostics, Queensland, Australia) was used for sera for fatal case confirmations. The IgG-ELISA was previously described by Miagostovich [30] and was performed to characterize the immune response as primary or secondary. The Platelia™ Dengue NS1 Ag-ELISA kit (Biorad Laboratories, Hercules, CA, USA) was used according to the manufacturer’s instructions. For the analysis by molecular techniques, the viral RNA was extracted from samples using the QIAamp Viral RNA Mini kit (Qiagen, Hilden, Germany) following the manufacturer’s instructions. RT-PCR for detecting and typing DENV was performed as described previously by Lanciotti [31].

After confirmation by at least one of the laboratorial methods performed, a total of 80 non-fatal and fatal dengue cases, representing the four serotypes, were randomly selected (DENV-1, *n* = 20 [10 non-fatal and 10 fatal]; DENV-2, *n* = 20 [10 non-fatal and 10 fatal]; DENV-3, *n* = 20 [10 non-fatal and 10 fatal] and DENV-4, *n* = 20 [10 non-fatal and 10 fatal]).

### 2.3. NS1 Antigenemia Quantification

The NS1 antigenemia quantification was performed as previously described by Heringer [32]. Briefly, a standard NS1 antigen curve (ng/mL) based on an equation (y = 1.321x + 0.1271) with *R*^2^ = 0.9542 was used and established using synthetic NS1 proteins (Native Antigen Company, Oxforshire, UK) corresponding to the NS1 of DENV-1 (Nauru/Western Pacific/1974), DENV-2 (Thailand/16681/84), DENV-3 (Sri Lanka D3/H/IMTSSA-SRI/2000/1266) and DENV-4 (Dominica/814669/1981) with 10-fold dilution. All samples were tested between days 1 to 8 after the disease onset.

### 2.4. Real Time RT-PCR (qRT-PCR) Assay for Viremia Quantification

DENV viremia quantification was estimated by using the quantitative qPCR system Taqman (PE Applied Biosystems, Foster City, CA, USA) according to the protocol described by Johnson [33].

### 2.5. Statistical Analysis

Statistical analysis was performed using Graphpad PRISM version 6 (GraphPad Software, La Jolla, CA, USA). The *T* and Analysis of variance (ANOVA) tests were used to evaluate the significance of the variable categories. Statistical significance was considered when *p*-value was <0.05.

## 3. Results

### 3.1. Analysis of NS1 Antigenemia

**Figure 1:**
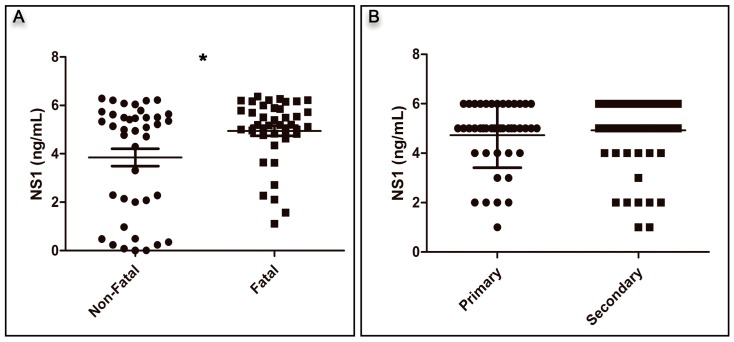

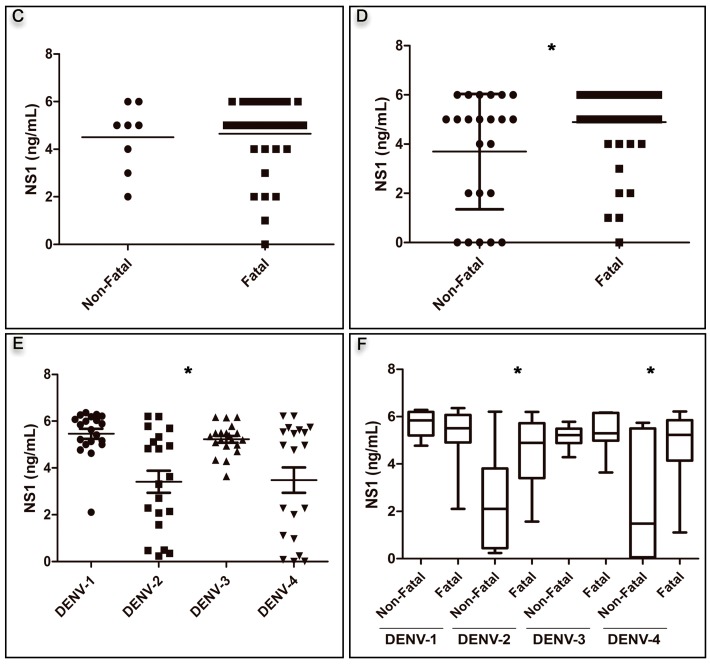
Non-structural protein 1 (NS1) antigenemia (**A**) according to the disease outcome (non-fatal: *n* = 40; fatal: *n* = 40), * *p* < 0.01 using *t* test; (**B**) in the overall analysis of the type of infection (primary: *n* = 24 versus secondary: *n* = 45) using *t* test; (**C**) in the analysis of primary non-fatal (*n* = 8) versus fatal (*n* = 16) cases by *t* test; (**D**) in the secondary non-fatal (*n* = 23) and fatal (*n* = 22) cases, * *p* < 0.01 using *t* test; (**E**) in the analysis of DENV serotype regardless of the disease outcome (*n* = 80) by ANOVA test * *p* < 0.01; (**F**) in the analysis of the distinct DENV serotypes according to the disease outcome (fatal: *n* = 10 and non-fatal/serotype) using *t* test. For DENV-2, fatal versus non-fatal cases, * *p* < 0.01, and for DENV-4 fatal versus non-fatal cases, * *p* < 0.01.

Overall, the NS1 antigenemia was significantly higher in dengue fatal cases when compared to non-dengue fatal ones (*p* = 0.01), Figure 1A. Regardless of the disease outcome, the average of NS1 levels in primary and secondary cases was similar (4.72 ng/mL and 4.92 ng/mL, respectively), Figure 1B. Furthermore, no differences were observed in NS1 levels between non-fatal and fatal primary cases (4.50 ng/mL and 4.65 ng/mL, respectively), Figure 1C. However, fatal cases from secondary infections showed significantly higher NS1 levels than non-fatal ones (4.89 ng/mL versus 3.69 ng/mL, *p* = 0.012), Figure 1D.

The overall analysis of the distinct DENV serotypes showed that, regardless of the disease outcome, DENV-1 showed significantly higher NS1 antigenemia, followed by DENV-3, DENV-4, and DENV-2 (*p* < 0.001; Figure 1D, Table 1).

In DENV-2 and DENV-4 fatal cases, the NS1 levels were significantly higher than those observed in non-fatal cases (*p* = 0.013 and *p* = 0.018, respectively, Figure 1F, Table 2). Furthermore, despite the high NS1 levels observed in DENV-1 and DENV-3, no differences were observed between fatal and non-fatal cases with those serotypes (Figure 1F, Table 2).

No differences were observed in the NS1 antigenemia of the different DENV serotypes between the types of infection (primary and secondary). The DENV-1 primary fatal cases had an average NS1 antigenemia of 4.80 ng/mL, and the secondary fatal cases had an average value of 5.66 ng/mL (*p* = 0.248). When DENV-2 fatal cases were analyzed by immune response, also no differences in the average of NS1 levels were observed (3.60 ng/mL and 3.30 ng/mL, respectively, *p* = 0.816). DENV-3 primary fatal cases had an average of 5.04 ng/mL of NS1, while the secondary fatal cases had a value of 5.16 ng/mL (*p* = 0.682). Because of an inadequate sample volume, only two DENV-4 fatal cases could be characterized according to the patient’s immune response, and the mean NS1 level was 5.50 ng/mL.

### 3.2. Analysis of Viral RNA Quantification

**Figure 2:**
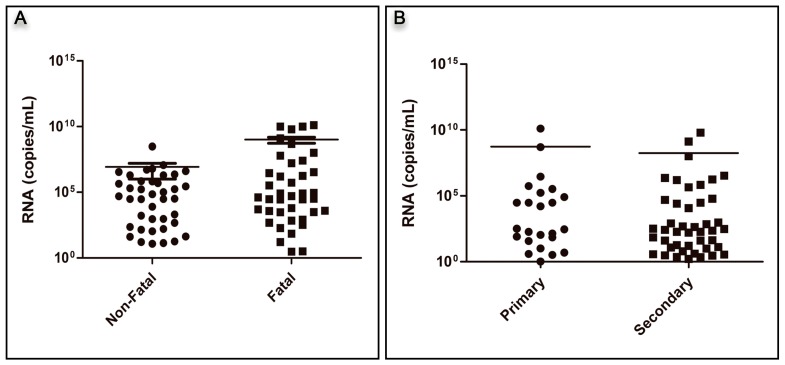

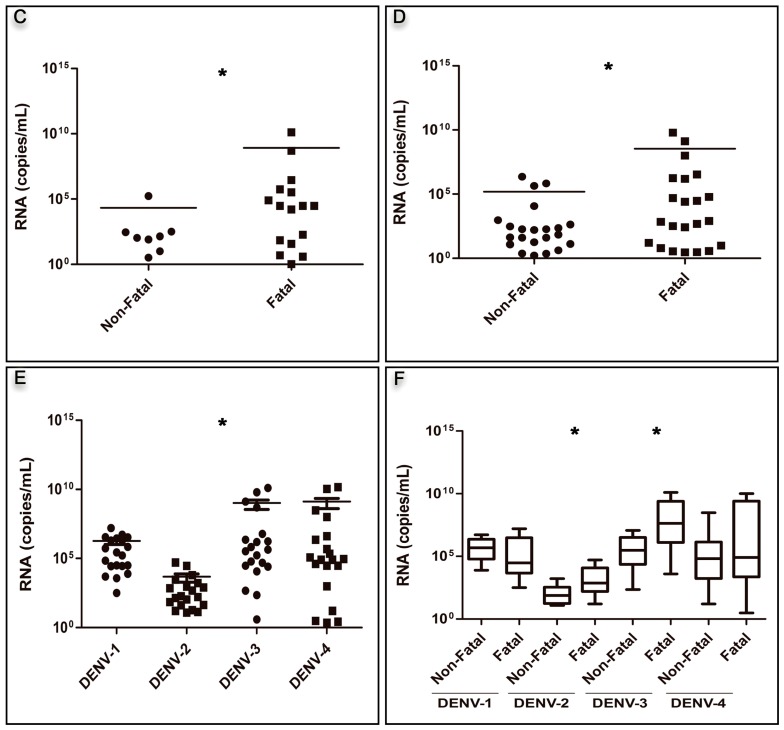
RNA viral load quantification by qRT-PCR (**A**) according to the disease outcome (non-fatal: *n* = 40; fatal: *n* = 40) *p* = 0.06 by using *t* test; (**B**) by type of infection (primary: *n* = 24 versus secondary: *n* = 45) using *t* test; (**C**) in primary non-fatal (*n* = 8) versus fatal (*n* = 16) cases, * *p* < 0.01 using *t* test; (**D**) in secondary non-fatal (*n* = 23) versus fatal (*n* = 22) cases, * *p* < 0.01 using *t* test; (**E**) in the analysis of DENV serotype regardless of the disease outcome (*n* = 80), * *p* < 0.01 by ANOVA test; (**F**) in the analysis of the distinct DENV serotypes according to the disease outcome (fatal: *n* = 10 and non-fatal/serotype) using *t* test. For DENV-2 fatal versus non-fatal cases, * *p* < 0.01, and for DENV-3 fatal versus non-fatal cases, * *p* < 0.01.

Overall, dengue fatal cases had a higher viral load than the non-fatal cases, but the difference was not statistically significant (*p* = 0.066), Figure 2A. No difference was also observed when the viremia from primary cases was compared with those from secondary ones (1.69 × 10^8^ copies/mL and 5.46 × 10^8^ copies/mL, respectively), regardless of the disease outcome (Figure 2B). However, in both primary and secondary fatal cases, the mean viremia was significantly higher than that observed in non-fatal primary and secondary cases (8.19 × 10^8^ copies/mL versus 2.13 × 10^4^ copies/mL, *p* = 0.0174, for primary and 3.45 × 10^8^ copies/mL versus 1.48 × 10^5^ copies/mL, *p* = 0.0048 for secondary), Figure 2C,D.

The overall analysis according to the distinct serotypes showed that DENV-3 and DENV-4 had significantly higher mean viremia compared to DENV-1 and DENV-2 (*p* = 0.001; Table 1; Figure 2E). However, considering the disease outcome, DENV-2 and DENV-3 fatal cases had significantly higher mean viral load compared to the non-fatal cases with the same serotype (*p* = 0.018 and *p* = 0.011, respectively), Table 2, Figure 2F.

Furthermore, when analyzing only the fatal cases, no differences were observed among primary and secondary cases of the distinct DENV serotypes. Primary DENV-1 fatal cases had a mean viral load of 5.80 × 10^6^ RNA/mL, and the secondary fatal cases of 8.60 × 10^5^ copies/mL (*p* = 0.298). DENV-2 mean viremia was 6.05 × 10^3^ copies/mL for the primary fatal cases and 5.22 × 10^3^ copies/mL for the secondary ones (*p* = 0.969). The mean viral load for DENV-3 fatal cases was 3.22 × 10^9^ copies/mL for primary and 1.25 × 10^9^ copies/mL for secondary fatal ones (*p* = 0.590). For DENV-4, no comparison was performed, as only one primary fatal case with 8.00 × 10^4^ copies/mL and two secondary fatal cases with a mean viral load of 5.00 × 10^7^ RNA/mL were available.

Cases representative of DENV-1 to 4 (*n* = 18) were previously sequenced by our group for genotype characterization, and Genotypes V, Southeast Asia, Genotype III, and Genotypes I and II were characterized for DENV-1, DENV-2, DENV-3, and DENV-4, respectively.

## 4. Discussion

Some viral proteins are described to be involved in viral disease pathogenesis, and the NS1 protein has been shown to be a marker of dengue disease severity [18,22,23,34,35,36]. Moreover, despite NS1 interaction with the complement system [23] and its involvement in the production of inflammatory and immunosuppressive cytokines by inducing immune cells [37,38], it is also suggested that NS1 induces endothelial hyperpermeability in vitro and vascular leak in vivo [24,25,39].

In this study, we observed that NS1 levels in fatal cases were higher than those in non-fatal ones (Figure 1A), and several studies have shared the same observation. An early study by Libraty [18] described the correlation between high circulating levels of NS1 and the development of severe dengue and was corroborated by similar observations in Thailand [23]. While analyzing Brazilian DENV-1 and DENV-4 cases, Allonso [22] observed increased NS1 levels in severe cases when compared to classic dengue. De la Cruz-Hernandez [35] reported that patients with dengue hemorrhagic fever (DHF), infected by DENV-1 showed higher levels of circulating NS1 than those with dengue fever (DF). On the other hand, the study by Duong [16] during an epidemic in Cambodia in 2006 and 2007, showed that NS1 levels significantly correlated with viremia, but a low NS1 ratio was associated with a more severe disease. In Finland, the levels of NS1 antigenemia from travel-acquired dengue cases were not associated with hospitalization [40]. A recent study in Colombia reported the correlation between high circulating levels of NS1 and the development of disease severity in children infected by DENV-1, DENV-2, and DENV-3 [41].

In this study, DENV-1 exhibited higher levels of NS1, followed by DENV-3, DENV-4, and DENV-2 (Table 2), corroborating previous observations [22,42,43]. Furthermore, Chau [42] found that NS1 levels were significantly higher in DENV-1- and DENV-3-infected infants under 18 months of age than in those infected by DENV-2, and also reported increased severity in DENV-3 cases; however, no correlation with viremia was observed. In Vietnam, a study analyzing the kinetics of plasma viremia and soluble NS1 in dengue-infected children reported higher NS1 levels in DENV-1 cases than in DENV-2 ones [43]. Considering the distinct serotypes and the disease outcome, it was shown here that DENV-2, DENV-3, and DENV-4 fatal cases presented higher NS1 antigenemia than non-fatal ones. Despite their non-significance, DENV-1 cases presented an opposite profile (Figure 1F).

Overall, the NS1 magnitude did not vary by type of infection, and no differences were observed in NS1 antigenemia between primary and secondary infections. In contrast, Perdomo-Celis [41] and De la Cruz-Hernandez [35] described that patients presenting primary infections had higher NS1 levels than those with secondary ones.

The NS1 persistence and antigenemia were previously shown to depend on the infecting DENV serotype [16,44], and patients presenting a persistent antigenemia (>5 days of illness) were more likely to develop a more severe disease [36]. However, in our study, the persistence of NS1 antigenemia was not addressed because of the nature of our sampling (convenience samples).

Overall, the fatal cases studied here had a higher viral load than the non-fatal ones, but the difference was not statistically significant (Figure 2A). Several studies of DENV viral quantification published previously showed a correlation between the amount of viral particles and increased disease severity [19,21,45,46,47,48]. In Brazil, studies with DENV-2 and DENV-3 [19,21] reported higher viremia in fatal cases and corroborated the observations made in Thailand on DENV-1 and DENV-2 and in Taiwan on DENV-3 [45,47].

Regardless of the disease outcome, no difference was also observed when the viremia from primary cases was compared to that from secondary ones (Figure 2B). On the other hand, the viremia levels were higher in fatal cases from both primary and secondary infections (Figure 2C,D). Tricou [20] observed that the peak in viremia was significantly observed less frequently during secondary than primary infections for all disease outcomes. In contrast, Perdomo-Celis [41] observed that viremia of primary infections was higher than in patients with secondary infections.

In a pediatric cohort in India, severe dengue was observed in primary and secondary infections; however, no association between viral load and disease severity was reported, despite a correlation with prolonged thrombocytopenia and delayed recovery [49]. This is in agreement with previous observations that showed no differences in viral RNA levels in children with DHF and classic dengue fever in the past [50].

In this study, DENV-4 and DENV-3 had a higher viremia, followed by DENV-1 and DENV-2 (Figure 2E). Tricou [20] similarly demonstrated that DENV-1 infections had higher viremia levels than DENV-2 infections, and, recently, Perdomo-Celis [41] reported that viremia of DENV-1, DENV-2, and DENV-3 were greater in severe cases than in cases without and with warning signs. However, De la Cruz-Hernandez [35] reported that classic DENV-1 and DENV-2 patients presented significantly higher levels of viremia when compared to the DHF ones. Here, all serotypes had higher viremia in fatal cases than in non-fatal ones, but these differences were statistically significant only for DENV-2 and DENV-3 serotypes (Figure 2F).

## 5. Conclusions

Dengue pathogenesis is multifactorial, but viral components, such as secreted NS1 and high viral load, may influence the disease outcome. Therefore, the complex interaction between viral and host immunological determinants should still remain the subject of many studies. In this study, it was observed that, irrespective of the infecting DENV serotype, viremia and NS1 levels were higher in fatal cases than in non-fatal ones, therefore suggesting these parameters as potential biomarkers for increased dengue severity. However, one cannot disregard a patient's ability to resolve the infection or, conversely, to succumb to it and evolve to death, and the factors involved in the latter, such as the availability of adequate and prompt assistance.

## Figures and Tables

**Table 1:** Overall NS1 antigenemia and RNA viral load according to the distinct DENV serotypes, independent of the disease outcome.

DENV Serotype	*n*	NS1 Antigenemia (Mean, ng/mL)	*p*	RNA Quantification (Mean, Copies RNA/mL)	*p*
Dengue 1	20	5.46	<0.001	1.83 × 106	=0.001
Dengue 2	20	3.41		4.73 × 103	
Dengue 3	20	5.23		1.03 × 109	
Dengue 4	20	3.48		1.30 × 109	

**Table 2:** NS1 antigenemia and RNA viral load according to the distinct DENV serotype and disease outcome.

Serotype	Disease Outcome	*n*	NS1 Antigenemia (mean, ng/mL)	*p*	RNA Quantification (mean, RNA Copies/mL)	*p*
Dengue 1	Non-Fatal	10	5.69	=0.285	1.38 × 10^6^	=0.266
	Fatal	10	5.27		2.29 × 10^6^	
Dengue 2	Non-Fatal	10	2.92	=0.013	3.13 × 10^1^	=0.018
	Fatal	10	4.53		9.15 × 10^3^	
Dengue 3	Non-Fatal	10	5.16	=0.663	2.15 × 10^6^	=0.011
	Fatal	10	5.29		2.06 × 10^9^	
Dengue 4	Non-Fatal	10	2.24	=0.016	3.04 × 10^7^	=0.731
	Fatal	10	4.73		2.01 × 10^9^	
